# Genome-Wide Identification of *S1fa* Transcription Factors in *Brassica napus* and Screening of Key Genes *BnaS1fa9* and *BnaS1fa10* Responsive to Salt, Heat and Cold Stresses

**DOI:** 10.3390/plants15121808

**Published:** 2026-06-12

**Authors:** Ruisheng Qi, Min Mi, Chunmiao Xu, Qingfan Guo, Yun Dong, Jingjing Chen, Jianye Wei, Renmei Dang, Zhaonan Wu, Bo Dong, Huizhen Ma, Zhiyang Ma

**Affiliations:** 1 Agricultural Technology Extension Service Center of Linxia Hui Autonomous Prefecture, Linxia 731100, China; qiruisheng163@163.com (R.Q.); lxznjfwzx@163.com (Q.G.); chjj6210778@163.com (J.C.); lxsmawen@163.com (H.M.); 2 National Key Laboratory of Crop Genetics & Germplasm Innovation and Utilization, Key Laboratory of Biology and Genetic Improvement of Horticultural Crops (East China), Ministry of Agriculture and Rural Affairs of China, Engineering Research Center of Germplasm Enhancement and Utilization of Horticultural Crops, Ministry of Education of China, Nanjing Agricultural University, Nanjing 210095, China; mm@njau.edu.cn; 3 Economic Crops Technology Extension Station of Anding District, Dingxi 743000, China; gslz0721@163.com; 4 Gansu Academy of Agricultural Sciences, Lanzhou 730070, China; dongyungs@163.com (Y.D.); dongbobby@163.com (B.D.); 5 Linxia Hui Autonomous Prefecture Academy of Agricultural Sciences, Linxia 731100, China; 15393028989@163.com (J.W.); 18394105722@163.com (R.D.); 17752268701@163.com (Z.W.)

**Keywords:** *Brassica napus* L., *S1Fa* gene family, transcription factor, abiotic stress, heat stress, reproductive development

## Abstract

*Brassica napus* reproductive development and abiotic stress tolerance are critical for yield and quality, and characterizing key transcription factor families is vital for molecular breeding. Here, based on the *B. napus* cv. Darmor-bzh V5 reference genome, we systematically identified and analyzed the *BnaS1fa* gene family, uncovering 12 members. Their encoded proteins are mostly small, alkaline, stable, and hydrophilic, with a few having ultra-long structures. Phylogenetic analysis clustered them into three subfamilies; conserved motif and gene structure analyses revealed high overall family conservation with partial member differentiation. Promoter *cis*-acting element analysis showed enrichment in light, hormone, and stress-responsive elements. Chromosomal localization and intraspecific collinearity analyses indicated the family mainly derived from homologous fragment retention in A and C subgenomes. Transcriptome data demonstrated high *BnaS1fa* expression in late seed and silique development, with prominent heat stress responses. RT-qPCR, subcellular localization and transcriptional activity assays confirmed *BnaS1fa9* and *BnaS1fa10* as nuclear-localized transcription factors with heat stress-induced expression. This study elucidates *BnaS1fa* molecular characteristics and its potential roles in reproductive development and heat stress response, providing candidate genes for *B. napus* stress-resistant molecular breeding. Further functional validation of these key genes will facilitate the dissection of their precise regulatory mechanisms governing heat stress tolerance and reproductive growth, which can be ultimately applied to advance the genetic improvement of rapeseed stress resistance and yield performance.

## 1. Introduction

*B. napus* L. is one of the most important oilseed crops and potential energy plants worldwide. Its yield and quality are not only determined by vegetative growth, but also tightly regulated by the coordination of reproductive development and adaptive responses to various abiotic stresses, including heat, drought, and salinity. With ongoing global climate change, abiotic stresses have become increasingly severe constraints on the yield stability and production efficiency of *B. napus*. Therefore, identifying key regulatory factors and their molecular mechanisms underlying reproductive development and stress adaptation is critical for molecular breeding of high-yield and stress-tolerant rapeseed varieties [1,2,3,4].

Transcription factors (TFs) serve as core regulators in plant gene expression networks [5,6,7,8,9]. By binding to specific *cis*-acting elements in the promoters of target genes, they precisely modulate multiple biological processes, including growth, development, and stress responses [10,11,12,13,14,15,16]. Among these, transcription factor families, which are typically small in size or have relatively few members, are often characterized by compact structures, rapid responsiveness, and high regulatory efficiency. It should be noted, however, that exceptions exist; for example, the peanut genome contains 126 S1Fa-like TFs [17]. During long-term evolution, many small transcription factor families have formed conserved but functionally diverse regulatory modules that enable plants to cope with environmental fluctuations, making them particularly attractive targets for genetic improvement. It should be noted, however, that large families such as the *NAM* family, which includes more than 100 members in rice and *Arabidopsis* [3], can also play central roles in stress adaptation and crop improvement.

The *S1Fa* (S1F-binding factor) family represents a group of plant-specific small transcription factors characterized by a conserved DNA-binding domain and nuclear localization signal [1,18,19]. Initially identified as a repressor of plastid gene transcription in spinach, S1Fa proteins target conserved *cis*-elements in the promoters of photosynthesis-associated genes such as *rbcS* and *cab* [20]. Functional studies in multiple species have revealed that *S1Fa* genes participate in diverse biological processes, including photomorphogenesis, flowering time control, drought resistance, ROS scavenging, and seed maturation. For instance, *OsS1Fa1* enhances drought tolerance by activating stress-responsive genes in rice; *PtS1Fa2* improves drought resistance by reducing ROS accumulation in *Populus trichocarpa* [1,21]. Recent transcript evidence also indicates that several S1Fa-like genes in *B. napus* are rapidly induced under salt stress, implying their potential roles in early stress signal perception and transduction [22].

*B. napus* is an allotetraploid species derived from the natural hybridization of *Brassica rapa (AA)* and *Brassica oleracea (CC)*. Its genome has undergone recurrent whole-genome duplication and structural rearrangement events, leading to extensive expansion and functional divergence of many gene families [23,24,25]. Although the biological importance of the *S1Fa* family has been validated in several model plants, a comprehensive genome-wide characterization of the *S1Fa* family in *B. napus*—including gene identification, phylogenetic relationships, gene structure, *cis*-element distribution, and expression divergence—remains lacking.

In this study, we performed a genome-wide identification and analysis of the *BnaS1fa* gene family based on the Darmor-bzh v5 reference genome [26]. We analyzed gene structures, chromosomal locations, phylogenetic relationships, *cis*-acting regulatory elements, tissue-specific expression patterns, and expression responses to multiple abiotic stresses. Our results reveal the molecular characteristics and potential functions of *BnaS1fa* genes, and provide valuable candidate genes for further functional studies and stress-resistance molecular breeding in *B. napus*.

## 2. Results

### 2.1. Identification and Characterization of the S1fa Gene Family in B. napus

The *BnaS1fa* gene family in *B. napus* was initially identified using the *S1fa* gene and protein sequences of *A. thaliana* (*AT2G37120*, *AT3G53370* and *AT3G09735*) via BLAST (version 2.12.0+) and HMMER software (version 3.3.2). After the initial identification of *S1fa* family genes using BLASTP (with known Arabidopsis S1fa proteins) and the Pfam HMM (PF04689), we constructed a custom hidden Markov model based on the multiple sequence alignment of the identified *B. napus* S1fa proteins. This species-specific model, together with the original PF04689 model, was then used to re-annotate and confirm the members of the BnaS1fa gene family. A total of 12 *BnaS1fa* transcription factors were ultimately identified in the *B. napus* genome.

Most BnaS1fa proteins were small-molecular-weight proteins (with the exception of *BnaA04g04820D* and *BnaC04g27370D*), with their amino acid lengths concentrated in the range of 70 to 76 residues. The isoelectric points (pI) of most of these small proteins ranged from 10.05 to 10.34, classifying them as alkaline proteins; only *BnaA04g04820D* (pI = 7.67) and *BnaC04g27370D* (pI = 7.98) were slightly alkaline neutral proteins. The instability indices of all BnaS1fa proteins varied from 21.34 to 39.69, all of which were below 40, indicating that these proteins possessed good stability in vitro. In addition, all BnaS1fa proteins exhibited hydrophilic properties overall (Table 1).

### 2.2. Phylogenetic Analysis of BnaS1fa Genes

**Figure 1:**
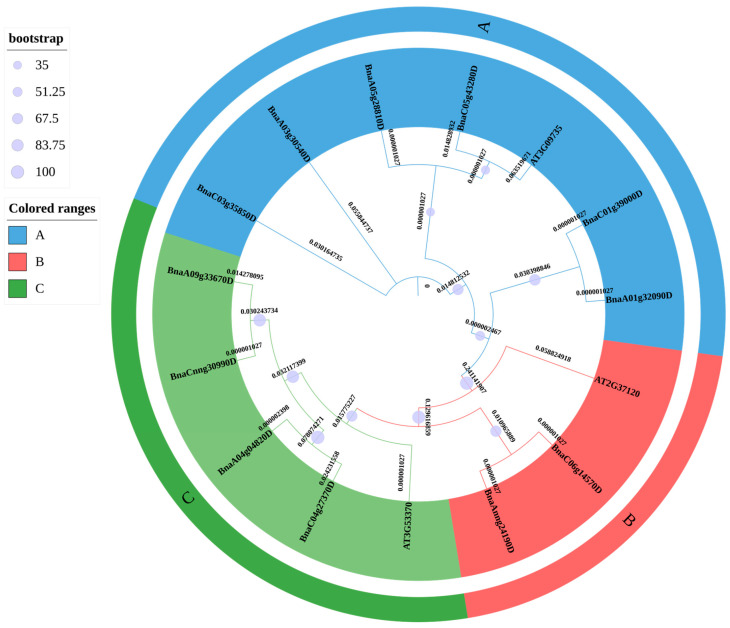
Phylogenetic tree of the *S1fa* genes in *B. napus* and *A. thaliana.* The circular phylogenetic tree was constructed using the neighbor-joining (NJ) method with 1000 bootstrap replicates, based on the full-length amino acid sequences of S1fa proteins from *B. napus* and *A. thaliana*. The three colored rings (A, B, C) represent the distinct phylogenetic subfamilies of *S1fa* genes.

Multiple sequence alignment of the identified *BnaS1fa* genes was performed using MUSCLE (version 3.8.31), followed by the construction of a phylogenetic tree via FastTree software (version 2.1.11) with the maximum likelihood (ML) method and JTT model to investigate the evolutionary relationships among *BnaS1fa* genes. Based on the genetic distance to *AtS1fas*, the *BnaS1fa* genes were divided into three subfamilies, designated as A, B and C. Subfamily A contained the largest number of *BnaS1fa* genes, while Subfamily B had the fewest; the two ultra-long BnaS1fa proteins exhibited the closest genetic relationship (Figure 1).

### 2.3. Motif Analysis, Gene Structure Analysis and Protein Tertiary Structure Prediction of BnaS1fa Genes

**Figure 2:**
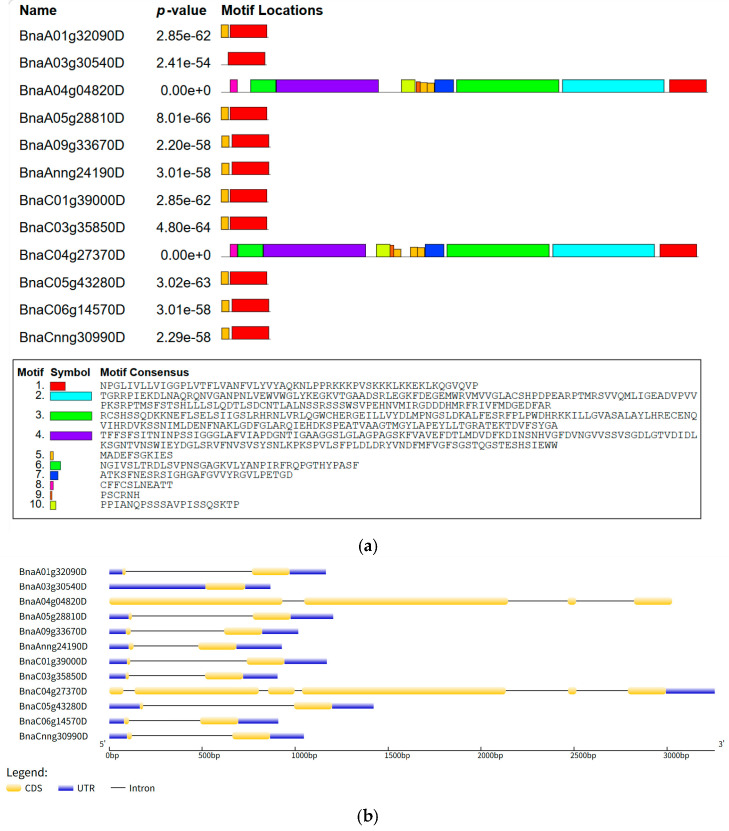

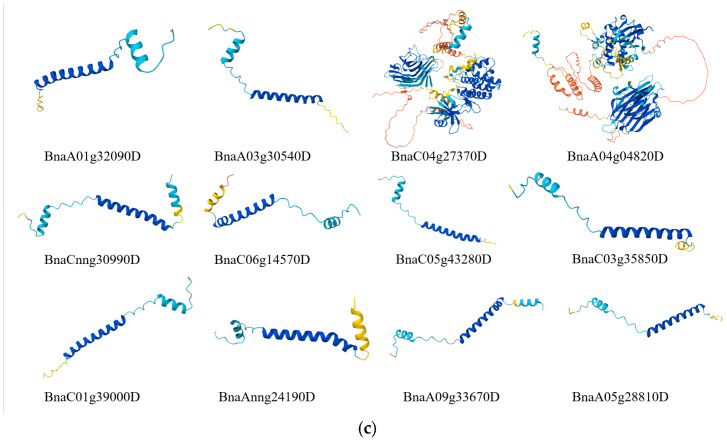
Comprehensive analysis of the *BnaS1fa* gene family in *B. napus*. (**a**) Conserved motif analysis of BnaS1fa proteins. The colored boxes represent conserved motifs (1–10), with specific motif sequences and consensus details provided in the lower table. (**b**) Gene structure analysis of *BnaS1fa* genes. The yellow blocks indicate coding sequences (CDS), blue blocks represent untranslated regions (UTRs), and black lines denote introns. The scale bar at the bottom indicates the genomic position in base pairs (bp). (**c**) Predicted tertiary protein structures of BnaS1fa members. The models are visualized using ribbon diagrams, with α-helices (blue) and random coils (yellow/yellow-red) representing the main secondary structural elements of the proteins.

Given the inextricable link between protein function, evolutionary relationships, and the types and composition of conserved motifs, we first analyzed ten conserved motifs in the 12 identified *BnaS1fa* proteins (Figure 2a). We found that the small *BnaS1fa* proteins basically contained only Motif 1 and Motif 10 (with the exception of *BnaA03g30540D*), and all *BnaS1fa* proteins contained Motif 10, indicating that Motif 10 may represent the core functional domain of BnaS1fa proteins. The two ultra-long BnaS1fa proteins (BnaA04g04820D and BnaC04g27370D) possessed extremely similar conserved motifs, including all Motif 1 to Motif 10, with a complex structure, which suggests that they may harbor more abundant functional domains and undertake more complex biological functions.

Analysis of the *BnaS1fa* gene structures (Figure 2b) showed that most of the small conserved structural genes had a total length of approximately 1000–1200 bp with a compact overall structure, containing only 1–2 CDS fragments. This indicates that these genes have a conserved structure and may play roles in basic physiological processes. In contrast, the two ultra-long genes had a complex structure with a total length of over 3000 bp (2–3 times that of the small genes), containing multiple CDS fragments and longer UTRs. The conserved structure of the small genes suggests that they may retain the core functions of the ancestral genes, while the complex structure of the long genes may have acquired new functions through gene duplication and domain insertion.

Multiple sequence alignment of the full-length proteins, including the two unusually long candidates (BnaA04g04820D and BnaC04g27370D), with other S1fa family members showed that all proteins share a highly conserved C-terminal domain (Figure S1). The extended N-terminal sequences of the two longer proteins do not affect the conserved family-specific domain structure, supporting their classification as members of the S1fa gene family.

The aforementioned gene structure analysis revealed a trend of functional differentiation within the family. To verify this inference, we predicted the spatial conformations of all *BnaS1fa* proteins using AlphaFold 3, thereby linking the linear structure of genes to the tertiary functional structure of proteins. The results of tertiary structure prediction demonstrated that the small proteins featured a core structure composed of 1–2 α-helices, with short random coils attached to both ends; they lacked complex folding or domain assembly, and presented an overall linear or slightly curved strip-like conformation. In comparison, the ultra-long complex proteins (*BnaA04g04820D* and *BnaC04g27370D*) consisted of alternating arrangements of multiple α-helices and β-sheets, with abundant random coils and loop structures on their surface, forming a tightly folded spherical or elliptical conformation that contained multiple functional domains (Figure 2c).

### 2.4. Prediction of Cis-Acting Elements in the Promoters of BnaS1fa Genes

**Figure 3:**
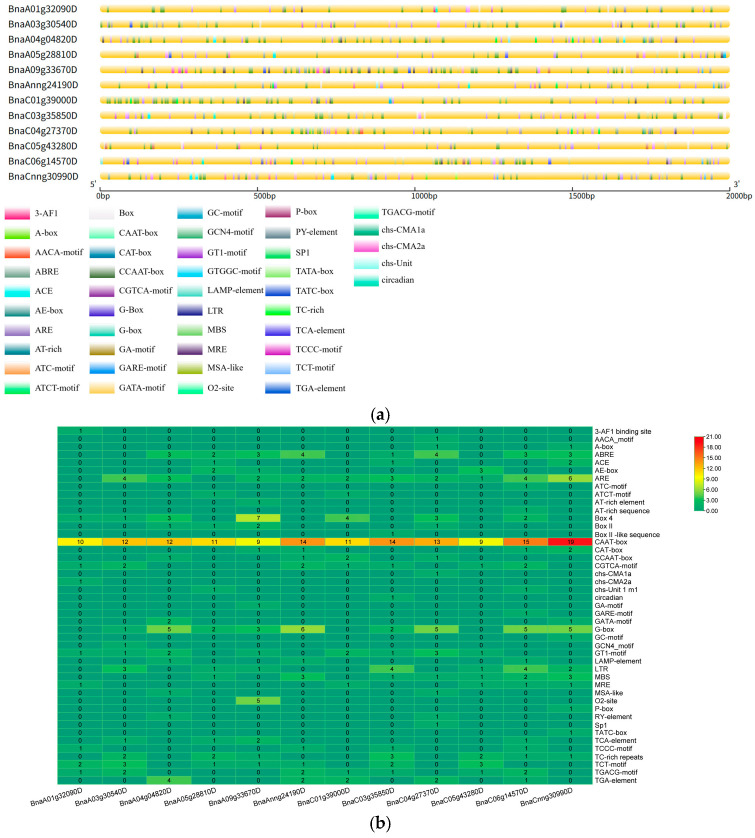
Analysis of *cis*-acting elements in the promoters of *BnaS1fa* genes in *B. napus*. (**a**) The 2000 bp promoter sequences upstream of the translation start codon (ATG) of all identified *BnaS1fa* genes were analyzed using the PlantCARE database. Different colored blocks represent distinct types of *cis*-acting elements, including basic transcriptional elements (e.g., TATA-box, CAAT-box), stress-responsive elements, hormone-responsive elements, and light-responsive elements. The detailed information of all *cis*-elements is provided in the Supplementary Materials. (**b**) Heatmap showing the number of each *cis*-acting element in the promoter of each *BnaS1fa* gene. The color scale on the right indicates the count of elements, with red representing high abundance and green representing low abundance.

As the core regulatory region for gene transcription, the composition and analysis of *cis*-acting elements in promoters constitute a key molecular basis for dissecting gene expression patterns and functional differentiation. To further elucidate the possible transcriptional regulatory mechanisms of the *BnaS1fa* gene family and their potential roles in abiotic stress responses, the 2000 bp upstream sequences of the 12 identified *BnaS1fa* members were extracted as promoter sequences for systematic prediction and analysis of *cis*-acting elements. The results showed that the promoters of *BnaS1fa* members mainly contained basic transcriptional elements, stress-responsive elements, hormone-responsive elements and light-responsive elements (Figure 3a). Combined with the heatmap of *cis*-acting elements in the promoters (Figure 3b), anaerobic responsive elements (ARE) were the most numerous (6 copies) in *BnaC06g14570D*, indicating that this gene may play an important role in hypoxic conditions; light-responsive elements (Box4 and G-box) were present in almost all promoters, suggesting that the whole family is generally regulated by light signals; stress and hormone-responsive elements (e.g., ABRE) were distributed in most genes, which demonstrates that *BnaS1fa* genes are extensively involved in abiotic stress and hormone signal responses. Notably, the TATC-box element was present only in the promoter of *BnaCnng30990D* and was absent from all other *S1fa* gene promoters analyzed (Figure 3b).

### 2.5. Chromosomal Localization and Intraspecific Collinearity Analysis of BnaS1fa Genes

**Figure 4:**
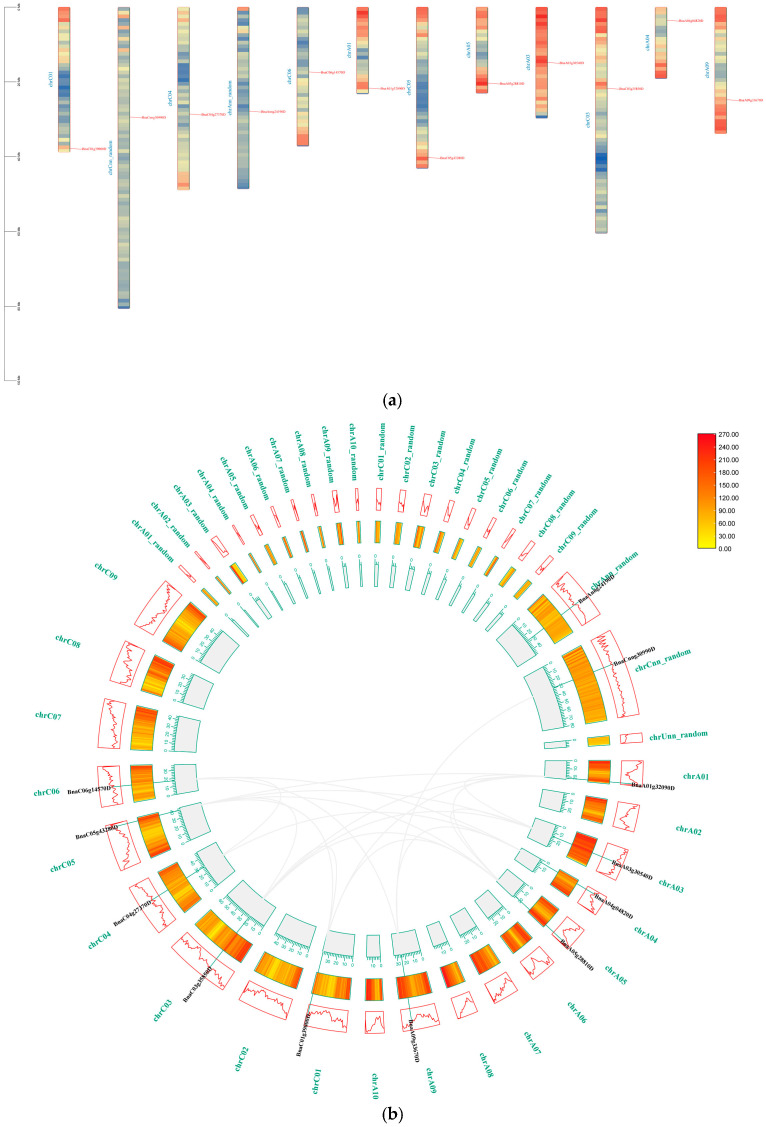
Chromosomal localization and collinearity analysis of the *BnaS1fa* gene family in *B. napus*. (**a**) Chromosomal distribution of *BnaS1fa* genes. The vertical bars represent the chromosomes of *B. napus*, with the positions of *BnaS1fa* genes marked on the corresponding chromosomes. (**b**) Collinearity analysis of *BnaS1fa* genes in the *B. napus* genome. The circular plot shows the collinear relationships between *BnaS1fa* genes, with the gray lines in the background representing all collinear gene pairs in the genome. The heatmap on the outer ring indicates the gene density distribution along each chromosome.

Chromosomal localization analysis is a core and fundamental approach for dissecting the origination, evolution, copy number variation and functional differentiation of gene families. To clarify the distribution characteristics and evolutionary patterns of the *BnaS1fa* gene family in the *Brassica napus* genome, chromosomal localization analysis was performed (Figure 4a). The results revealed that the family members were scattered across 10 distinct chromosomes and 2 random scaffolds, with no large-scale tandem clustering observed. This indicated that the expansion of this family was mainly dependent on whole-genome duplication or segmental duplication, rather than tandem duplication. Most genes were localized in the upper and middle regions of chromosomes, while only a few were mapped to the pericentromeric regions. This distribution pattern may be attributed to the recombination rate and selective pressure of chromosomes.

Intraspecific collinearity analysis was conducted for the *BnaS1fa* family genes (Figure 4b), and the results showed that most *BnaS1fa* genes had one-to-one or one-to-many collinear gene pairs between the A and C subgenomes.

### 2.6. Analysis of Tissue Expression Specificity and Expression Patterns Under Abiotic Stresses of BnaS1fa Genes

**Figure 5:**
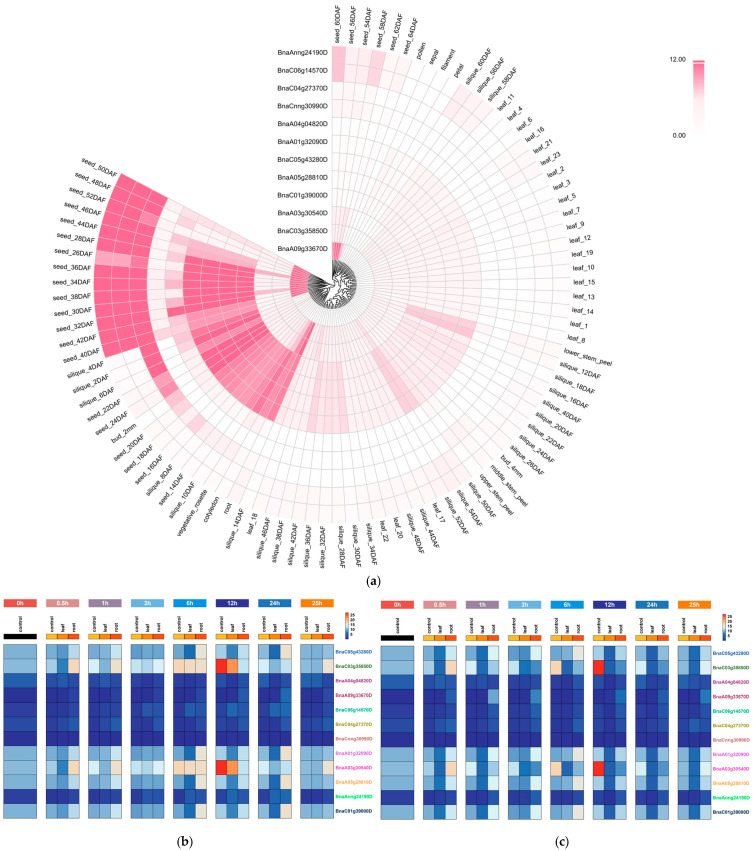

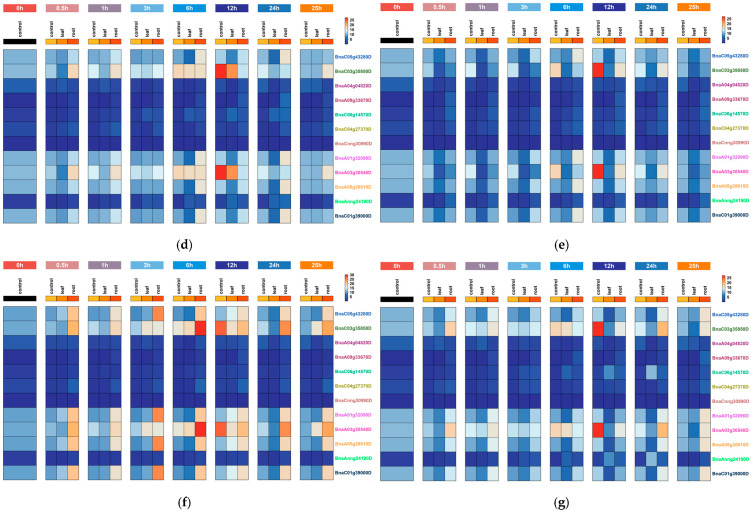
Expression heatmaps of the *BnaS1fa* gene family in *B. napus*. (**a**) Tissue-specific expression profile. (**b**) Expression under salt stress. (**c**) Expression under cold stress. (**d**) Expression under drought stress. (**e**) Expression under freezing stress. (**f**) Expression under heat stress. (**g**) Expression under osmotic stress.

Transcriptome data were used to analyze the expression profiles of *BnaS1fa* genes in different tissues (Figure 5a). We found that the *BnaS1fa* family genes exhibited markedly high expression in the late stage of seed development and silique development, with their expression levels peaking particularly at the seed-30DAF to seed-52DAF stages. This result indicated that the core functions of the BnaS1fa family genes may be closely associated with seed maturation and silique development. Interestingly, the expression of *BnaS1fa* genes was generally low in vegetative organs, which further confirmed that the functions of this family are biased toward the reproductive development stage.

Abiotic stresses (e.g., high temperature, drought, and high salinity) have long been the major environmental factors limiting the yield and quality of *B. napus* [27]. To investigate the roles of *BnaS1fa* genes in responding to abiotic stresses, transcriptome data were used to systematically analyze the stress-induced expression patterns of *BnaS1fa* family members (Figure 5b–g). Under salt stress, the expression of *BnaC03g35850D* and *BnaA03g30540D* was significantly downregulated, especially in roots. In the cold treatment group, most *BnaS1fa* genes showed no significant expression changes, and only a few genes (e.g., *BnaC03g35850D* and *BnaA03g30540D*) exhibited obvious downregulation; transcriptome data indicated that the *BnaS1fa* family had a weak response to cold stress. The expression patterns of *BnaS1fa* genes under salt and drought stresses were similar. Under heat stress, half of the *BnaS1fa* genes showed upregulated expression, and *BnaC03g35850D* and *BnaA03g30540D* displayed an extremely significant and strong expression at 6 h, demonstrating that the *BnaS1fa* family has a robust response to heat stress.

### 2.7. Validation of BnaS1fa Genes Responses to Abiotic Stresses by qPCR

**Figure 6:**
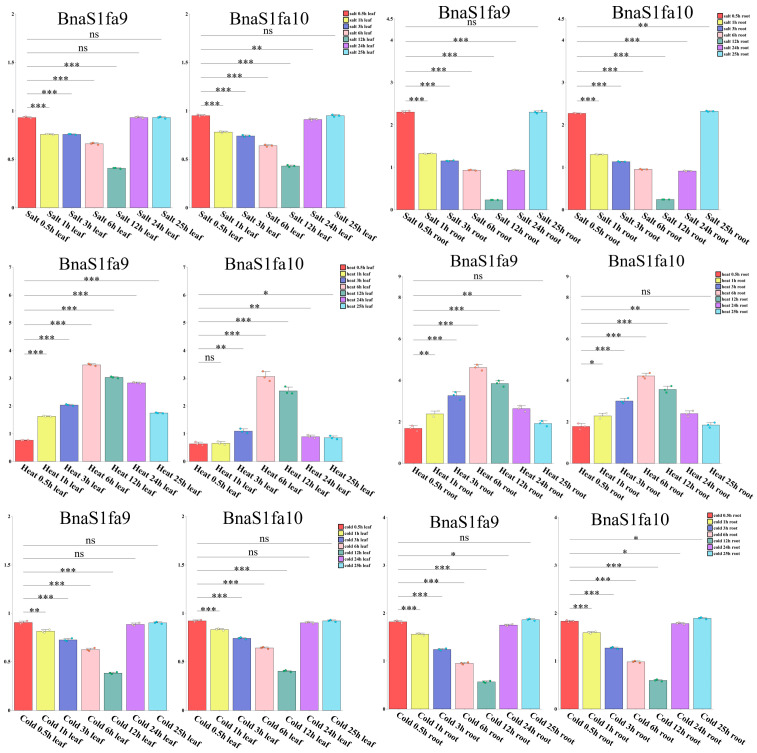
The expression patterns of *BnaS1fa9* and *BnaS1fa10* in *B. napus* leaves and roots under salt, heat, and cold stresses. Relative expression levels were determined by RT-qPCR at 0, 0.5, 1, 3, 6, 12, 24, and 25 h after continuous stress treatment. Untreated samples at each corresponding time point were used as controls to eliminate circadian rhythm interference. Cold stress was imposed at 4 °C, salt stress was treated with 200 mM NaCl, and heat stress was performed at 38 °C. All data are presented as mean ± standard deviation (SD) from three independent biological replicates (* *p* < 0.05, ** *p* < 0.01, *** *p* < 0.001, NS: not significant (*p* > 0.05) ). *BnaActin2* was used as the internal reference gene for expression normalization.

To validate the expression patterns of *BnaS1fa* family genes under different abiotic stresses as revealed by transcriptome data, and to further clarify the stress response specificity of core members (*BnaC03g35850D* and *BnaA03g30540D*), we selected the aforementioned key genes, along with time points and tissue samples from typical stress treatments (salt, cold and heat stress), to perform quantitative validation of their expression levels via real-time fluorescent quantitative PCR (RT-qPCR) (Figure 6). Based on their chromosomal locations, *BnaC03g35850D* and *BnaA03g30540D* were designated as *BnaS1fa10* and *BnaS1fa9*, respectively. Quantitative data demonstrated that the expression levels of *BnaS1fa9* and *BnaS1fa10* exhibited a high degree of similarity under different abiotic stresses.

Under salt stress, the expression levels decreased gradually with the extension of treatment time, reaching the lowest point at 12 h (only 15–20% of the initial level), and recovered in the late stage of treatment. This indicated that salt stress exerted a predominantly inhibitory effect on the expression of *BnaS1fa9* and *BnaS1fa10*. Under low-temperature stress, the expression levels dropped to a trough at 12 h and then rebounded. Interestingly, under heat stress, *BnaS1fa9* and *BnaS1fa10* displayed a distinctly different response pattern from that under salt or low-temperature stress: their expression was significantly upregulated in both roots and leaves, with the peak appearing at 6 h, and the upregulation amplitude was significantly higher than the responses under salt or low-temperature stress. This result indicated that heat stress is the strongest signal inducing the expression of *BnaS1fa9* and *BnaS1fa10*, and the responses of *BnaS1fa9* and *BnaS1fa10* to all stresses were more intense in roots than in leaves.

### 2.8. BnaS1fa9 and BnaS1fa10 Are Nuclear-Localized Transcription Factors

**Figure 7:**
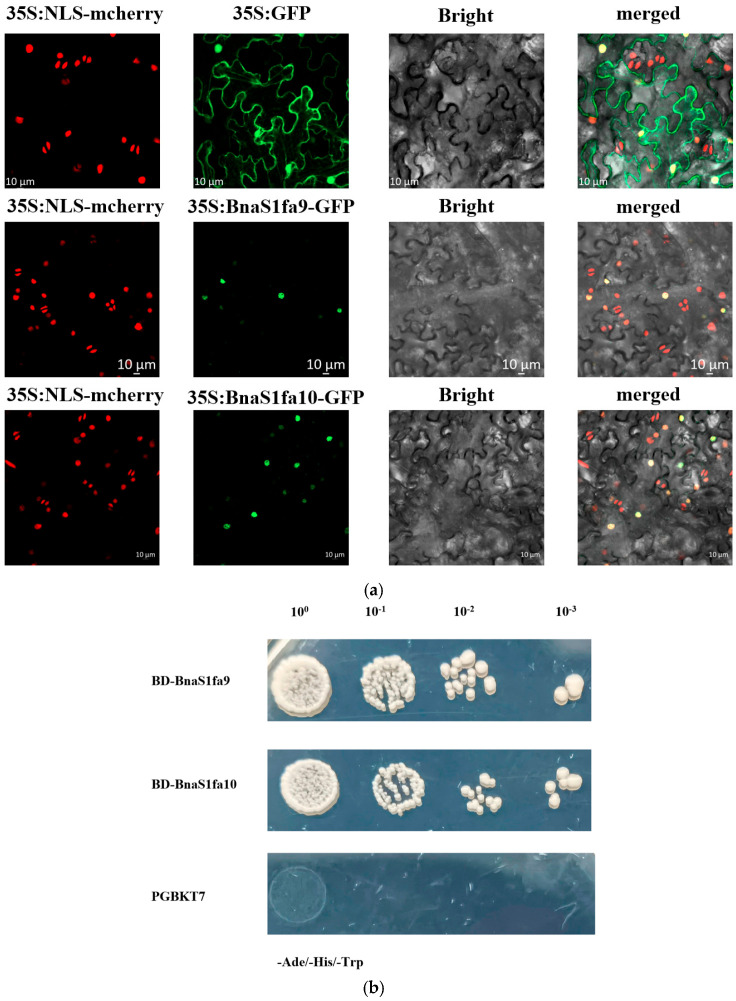
Functional analysis of *BnaS1fa9* and *BnaS1fa10*. (**a**) Subcellular localization of *BnaS1fa9* and *BnaS1fa10* in *Nicotiana benthamiana* leaves. The 35S:GFP empty vector was used as the control, and 35S:NLS-mCherry was used as the nuclear marker. The fluorescence signals (GFP, mCherry), bright-field images, and merged images are shown, with a scale bar of 10 μm. (**b**) Transcriptional activation activity assay of *BnaS1fa9* and *BnaS1fa10* in yeast. The BD fusion vectors (BD-*BnaS1fa9*, BD-*BnaS1fa10*) and the empty BD vector (pGBKT7, negative control) were transformed into yeast cells and cultured on SD/-Ade/-His/-Trp selective medium with 10-fold serial dilutions (10^0^, 10^−1^, 10^−2^, 10^−3^).

To further investigate the molecular functions of *BnaS1fa9* and *BnaS1fa10*, their subcellular localization was first determined via the tobacco transient expression system; the results showed that they were predominantly localized in the nucleus (Figure 7a). Subsequently, a yeast assay was performed to verify their transcriptional activity, with PGBKT7 serving as the negative control. The results demonstrated that yeast cells expressing BD-*BnaS1fa9* and BD-*BnaS1fa10* were able to grow on SD/-Ade/-His/-Trp medium, whereas PGBKT7-transformed yeast cells could not (Figure 7b). Collectively, these results confirm that *BnaS1fa9* and *BnaS1fa10* are nuclear-localized transcription factors.

## 3. Discussion

The *S1fa* gene family represents a group of highly conserved small transcription factors involved in plant growth, development and stress responses [1,22]. In this study, 12 *BnaS1fa* genes were systematically identified and characterized in *B. napus*. Combined analyses of phylogeny, gene structure, conserved motifs, *cis*-elements, collinearity and stress-responsive expression provided insights into the evolutionary conservation and potential functional diversification of the *S1fa* family in this allotetraploid species.

The core S1Fa domains of BnaS1fa proteins exhibit highly similar amino acid lengths and physicochemical properties, reflecting strong evolutionary conservation within the gene family. Their small molecular weights, alkaline isoelectric points and negative GRAVY values suggest that these proteins are hydrophilic nuclear transcription factors, consistent with previous reports in other plant species [1,21].

Gene structure and conserved motif analyses revealed high structural conservation among BnaS1fa members, particularly the universal presence of Motif 1, suggesting functional conservation within the family. However, several members containing additional conserved motifs may possess more diverse regulatory functions. In addition, the relatively simple exon–intron organization (mainly 1–2 introns) is consistent with the characteristics of rapidly responsive stress-related genes [28].

The multiple sequence alignment confirmed that the two unusually long proteins belong to the *S1fa* gene family, despite their extended N-terminal regions and overall structural differences compared to other family members. While they exhibit considerable variation in gene structure, non-core motifs, and predicted 3D conformation, their conserved core S1fa domains clearly place them within the family. This structural variation reflects the evolutionary plasticity of the *S1fa* family, where conserved core domains coexist with lineage-specific extensions and rearrangements. These N-terminal additions and structural differences may contribute to functional diversification, potentially modifying the regulatory or interaction properties of these two proteins relative to other family members.

*Cis*-element analysis revealed that *BnaS1fa* promoters contain numerous stress- and light-responsive elements, including ARE, Box4 and G-box motifs, suggesting potential involvement in environmental stress and light signaling pathways [29,30,31,32]. The diversity of cis-elements may contribute to the differential expression patterns of BnaS1fa genes under various conditions [33,34]. The presence of a TATC-box, a *cis*-acting regulatory element associated with gibberellin (GA) responsiveness, only in *BnaCnng30990D* may reflect a specific regulatory adaptation of this gene, potentially affecting its expression pattern under specific environmental or developmental conditions, particularly in response to GA signals. This regulatory divergence could contribute to functional specialization among the *S1fa* gene family members.

Collinearity analysis indicated that the expansion of the *BnaS1fa* family was mainly associated with allopolyploidization and duplication events. Most homologous gene pairs were retained between the A and C subgenomes, suggesting strong evolutionary conservation of this transcription factor family. Such retention patterns are consistent with previous studies showing that transcription factor genes are preferentially preserved after whole-genome duplication due to their regulatory importance [35,36].

In addition, duplicated gene pairs may contribute to functional redundancy and diversification during evolution [37,38,39].

The preferential expression of *BnaS1fa* genes during late seed and silique development suggests that they play important roles in reproductive processes. Similar developmental stage-specific expression patterns have been reported for transcription factors involved in seed maturation and reproductive organ development, where precise temporal regulation is essential [40].

The low expression levels in vegetative tissues further support the idea that *BnaS1fa* genes are not primarily involved in basic vegetative growth, but rather contribute to specialized developmental programs. This tissue-specific expression pattern reflects functional specialization and may indicate that S1fa proteins participate in transcriptional networks regulating reproductive development, possibly in coordination with hormonal or environmental signals.

The differential responses of *BnaS1fa* genes to various abiotic stresses highlight their functional specificity. Unlike many transcription factor families that exhibit broad-spectrum stress responsiveness, *BnaS1fa* genes showed relatively weak responses to salt, drought, freezing, and osmotic stresses. This suggests that they are not general stress regulators, but may instead function in specific signaling pathways.

In contrast, the strong induction under heat stress indicates that *S1fa* genes may play a specialized role in thermotolerance. Similar heat-responsive expression patterns have been reported for certain transcription factors that regulate heat shock proteins and other protective mechanisms [40]. Therefore, *BnaS1fa* genes may act as upstream regulators or modulators in heat stress signaling pathways, contributing to the adaptation of *B. napus* to high-temperature environments.

The consistent expression patterns of *BnaS1fa9* and *BnaS1fa10* under multiple stress conditions provide further evidence for the functional conservation of homeologous gene pairs. Their similar responses suggest that these genes have retained redundant or overlapping functions since the polyploidization event, which is consistent with the collinearity analysis.

However, the subtle differences in expression dynamics may indicate the early stages of functional divergence. Such partial redundancy is commonly observed in duplicated genes, allowing plants to maintain robustness while providing opportunities for evolutionary innovation.

Under salt and cold stress, the downregulation of *BnaS1fa9* and *BnaS1fa10* suggests that these genes may be negatively regulated or play a limited role in these stress responses. The stronger response observed in roots is consistent with the role of roots as primary sensors of environmental stress.

In contrast, their strong and rapid induction under heat stress, with peak expression at 6 h, indicates that they may function as early response genes in heat signaling pathways. This temporal expression pattern is characteristic of transcription factors that regulate downstream protective genes.

Notably, the stress response profiles of *BnaS1fa* genes differ substantially from those of *S1fa* homologs in Chinese cabbage (*Brassica rapa*) and rice (*Oryza sativa*). In Chinese cabbage, *S1fa* genes are significantly induced by heavy metal stresses (Hg, Cd) but repressed by salt stress [22]; in rice, *OsS1Fa1* is strongly upregulated by drought and positively regulates drought tolerance, while its homologs negatively modulate heat stress responses [21]. These interspecific divergences indicate that *S1fa* family genes have undergone functional differentiation during evolution. While the core conserved domains maintain their fundamental roles, the regulatory regions have diverged, leading to distinct stress response specificities across species. This functional divergence may reflect adaptive evolution to species-specific environmental pressures, with *BnaS1fa* genes evolving a specialized role in heat stress adaptation in allotetraploid rapeseed.

Taken together, these results suggest that *BnaS1fa* genes exhibit clear stress-type specificity, with a predominant role in heat stress responses rather than general stress adaptation. This specialization may reflect an evolutionary strategy to fine-tune plant responses to different environmental challenges.

The verification of transcriptional activation activity and nuclear localization provides important functional evidence supporting the predicted roles of BnaS1fa proteins as transcription factors. While sequence analysis suggested the presence of nuclear localization signals and conserved DNA-binding motifs, experimental validation confirms that these proteins are not only structurally consistent with transcription factors but are also functionally competent in regulating gene expression.

Although this study provides a comprehensive characterization of the BnaS1fa family, the downstream target genes and regulatory mechanisms of BnaS1fa proteins remain unclear. Future studies combining genetic analysis, protein–protein interaction assays and multi-omics approaches will help elucidate their biological functions, particularly in heat stress responses and stress adaptation in *B. napus*.

## 4. Materials and Methods

### 4.1. Identification of BnaS1fa Genes

The genome of *B. napus* was downloaded from BRAD [41] (http://brassicadb.cn/#/ (accessed on 2 February 2026)). To identify the *S1fa* genes in *B. napus*, we first retrieved the gene sequences and protein sequences of *A. thaliana S1fa* genes from The *A. thaliana* Information Resource (TAIR) database [42] (https://www.arabidopsis.org/ (accessed on 4 February 2026)), followed by obtaining the HMM model (PF04689) of the *S1fa* family from the Pfam website [43] (https://pfam.xfam.org/ (accessed on 4 February 2026)). The initial identification of *BnaS1fa* genes in *B. napus* was performed using BLAST (version 2.12.0+) [44] and HMMER (version 3.3.2) [45] software with the *S1fa* gene sequences and protein sequences of *A. thaliana* as queries. After taking the intersection of the results from both identification methods, a new model was reconstructed for the prediction of the *BnaS1fa* gene family. The final set of *BnaS1fa* genes was obtained by taking the intersection of the two rounds of identification results, ensuring the reliability and completeness of the identified gene family members. The ExPASy ProtParam tool [46] (http://www.expasy.org/ (accessed on 4 February 2026)) was used to predict the amino acid length, molecular weight (MW), theoretical isoelectric point (pI), instability index, and aliphatic index.

### 4.2. Construction of the BnaS1fa Phylogenetic Tree

The identified *BnaS1fa* genes were first subjected to multiple sequence alignment using MUSCLE. Subsequently, the phylogenetic tree was constructed via the maximum likelihood (ML) method with the JTT model using FastTree software (version 2.1.11) [47], and finally visualized and polished using iTOL [48] (https://itol.embl.de/ (accessed on 5 February 2026)). Based on the BLAST results, the identified family members were classified into different groups.

### 4.3. Motif Prediction and Gene Structure Analysis of BnaS1fa Genes

The MEME software (version 5.5.9) was employed to predict the motifs of the identified *BnaS1fa* genes, and a custom R script was used to generate motif sequence logo plots. Information on the gene family members was extracted from the *B. napus* genome annotation file, and the gene structure diagrams were constructed using the online GSDS tool [49] (https://gsds.gao-lab.org/index.php (accessed on 7 February 2026)).

### 4.4. Prediction of Cis-Acting Elements in the Promoters of BnaS1fa Genes

Based on the downloaded genome annotation file, the 2000 bp upstream sequence of the identified *BnaS1fa* genes was extracted as the promoter region for the prediction of *cis*-acting elements. Subsequently, the online tool PlantCARE (https://bioinformatics.psb.ugent.be/webtools/plantcare/html/ (accessed on 7 February 2026)) [50] was used to predict *cis*-acting elements in the extracted promoter sequences. After filtering the prediction results, the online GSDS tool [49] (https://gsds.gao-lab.org/index.php (accessed on 7 February 2026)) was employed for visual mapping of the *cis*-acting elements.

### 4.5. Chromosomal Localization of BnaS1fa Genes

The positions of the identified *BnaS1fa* genes on chromosomes and contigs were analyzed and visualized using TBtools (version 1.127) [51] based on the genome annotation file.

### 4.6. Tissue Expression Specificity and Heat Stress Expression Pattern Analysis of BnaS1fa Genes Based on Transcriptome Data

Transcriptome data of *BnaS1fa* genes, including tissue-specific expression profiles and expression data under abiotic stress (heat treatment), were retrieved from BnIR [52] (https://yanglab.hzau.edu.cn/BnIR (accessed on 10 February 2026)) for subsequent analysis. A custom R script was utilized to generate heat maps for the visualization of the expression patterns.

### 4.7. Plant Materials and Growth Conditions

*B. napus* cv. Darmor-bzh was used as experimental materials in this study. Seeds were surface-sterilized with 75% ethanol for 1 min and 1% sodium hypochlorite for 10 min, followed by 5 washes with sterile distilled water. Sterilized seeds were germinated on 1/2 Murashige and Skoog (MS) (Coolaber, Beijing, China) medium for 7 days, and then the uniform seedlings were transplanted into soil (a 1:1 mixture of peat and vermiculite) and grown in a controlled growth chamber with a 16 h light/8 h dark photoperiod, 22 °C constant temperature, 60% relative humidity, and 300 μmol·m^−2^·s^−1^ light intensity. Plants were watered regularly with Hoagland nutrient solution once a week to ensure sufficient nutrient supply, and leaf samples were collected at the 4–5 leaf stage for subsequent experiments.

### 4.8. RT-qPCR Analysis of BnaS1fa Expression Patterns Under Abiotic Stresses

Following abiotic stress treatments, 2–3 fully expanded leaves and approximately 0.1 g of root tissues (harvested from the root tip zone) were harvested from each sample for total RNA extraction. The expression levels of target genes were quantified via real-time quantitative PCR (RT-qPCR). The 20 μL RT-qPCR reaction system contained 10 μL of 2× TB Green Premix Ex Taq II, 0.4 μL of each primer (10 μM), 2 μL of cDNA template, and 7.2 μL of RNase-free water. The amplification program was 95 °C for 30 s, followed by 40 cycles of 95 °C for 5 s and 60 °C for 30 s, with a melting curve analysis to verify amplification specificity. *BnaActin2* was used as the internal reference for normalization. Relative gene expression levels were calculated via the 2^−ΔΔCT^ method. Gene expression was determined at 0 h (initial untreated control), 0.5 h, 1 h, 3 h, 6 h, 12 h, 24 h, and 25 h under continuous stress treatment, with untreated samples collected at each corresponding time point serving as the control to eliminate the interference of circadian rhythm on gene expression. Specifically, cold stress was performed at 4 °C, salt stress was applied with 200 mM NaCl, and heat stress was conducted at 38 °C. All data are presented as the mean ± standard deviation (SD) of three independent biological replicates. All primer sequences are provided in Supplementary Table S1.

### 4.9. Subcellular Localization

The plasmids pCAMBIA1302, pCAMBIA1302-*BnaS1fa9*, and pCAMBIA1302-*BnaS1fa10* were transformed into Agrobacterium tumefaciens competent cells (strain GV3101 (Coolaber, Beijing, China)). The transformed cells were cultured in liquid LB medium at 28 °C with shaking at 200 rpm for 16–24 h for subsequent use. Bacterial cells were harvested by centrifugation at 6000 rpm for 10 min and resuspended in an infiltration buffer (10 mM MES (Coolaber, Beijing, China), 10 mM MgCl_2_ (Coolaber, Beijing, China), and 150 μM acetosyringone [As] (Coolaber, Beijing, China)). The optical density at 600 nm (OD_600_) of the bacterial suspension was adjusted to 0.8. After standing at room temperature in the dark for 3–4 h, the suspension was infiltrated into *Nicotiana benthamiana* leaves carrying a mCherry-tagged nuclear localization marker. The infiltrated plants were cultured in the dark for 12 h, followed by light cultivation for 36–60 h. Using pCAMBIA1302 empty vector as the control, the fluorescent signals in tobacco leaves were detected and imaged using a high-resolution confocal laser scanning microscope (LSM900, Zeiss, Oberkochen, Germany).

### 4.10. Analysis of Transcriptional Activation Activity of Transcription Factors

The coding sequence (CDS) of *BnaS1fa9* and *BnaS1fa10* genes, with the stop codon removed, was inserted into the pGBKT7 vector. The recombinant vectors were then transformed into the yeast strain Y2HGold (Coolaber, Beijing, China), which was spread on a synthetic dropout (SD) medium lacking tryptophan (SD/-Trp) (Coolaber, Beijing, China) and cultured inverted in an incubator at 28 °C for 3–4 days. Yeast colonies were picked and diluted with sterile water to optical density at 600 nm (OD_600_) values of 0.2, 0.02, 0.002, and 0.0002. The diluted yeast suspensions were spotted onto SD medium deficient in adenine, histidine, and tryptophan (SD/-Ade/-His/-Trp) (Beijing LABLEAD Inc.), and the growth status was observed after incubation at 28 °C for 2–3 days.

## 5. Conclusions

In this study, we systematically identified 12 *S1fa* family genes in *B. napus* and comprehensively analyzed their physicochemical properties, phylogenetic relationships, gene structures, conserved motifs, promoter cis-elements, chromosomal distribution, collinearity, and spatiotemporal expression patterns. The *BnaS1fa* family was divided into three subfamilies with clear structural differentiation: most members were small, stably expressed, alkaline, and hydrophilic proteins with conserved motifs and compact gene structures, while two exceptionally long proteins exhibited complex domain compositions and tertiary structures.

Promoter analysis revealed abundant stress-, hormone- and light-responsive *cis*-elements, indicating extensive involvement in environmental adaptation. Expression profiles showed that *BnaS1fa* genes were predominantly expressed during seed and silique development, and strongly responded to heat, salt, drought, and osmotic stresses.

Two key members, *BnaS1fa9* and *BnaS1fa10*, were further validated by RT-qPCR and functional assays. Both were significantly induced by heat stress but inhibited by salt and cold stress, with stronger responses in roots. Subcellular localization and yeast transcriptional activation assays confirmed that they localize to the nucleus and possess transcriptional activation activity, acting as functional transcription factors.

Overall, this study provides a systematic overview of the *BnaS1fa* gene family and identifies *BnaS1fa9* and *BnaS1fa10* as important candidate genes involved in heat and abiotic stress responses in *B. napus*.

Moving forward, further research is needed to dissect the detailed regulatory mechanisms of these two transcription factors, including their specific binding sites, target genes, and interaction networks under stress conditions. Genetic transformation and field trials are also required to validate their functional roles in improving stress tolerance and agronomic performance in rapeseed. Ultimately, these findings may facilitate the development of stress-tolerant rapeseed varieties through marker-assisted breeding or genetic engineering, contributing to sustainable crop production under changing climates.

## Figures and Tables

**Table 1:** Physicochemical properties of the identified BnaS1fa proteins in *B. napus*.

Sequence ID	Number of Amino Acid	Molecular Weight	Theoretical pI	Instability Index	Aliphatic Index	Grand Average of Hydropathicity
BnaA01g32090D	73	8068.82	10.11	21.31	106.71	−0.149
BnaA03g30540D	70	7731.49	10.34	31.43	109.86	−0.169
BnaA04g04820D	758	82,257.48	7.67	39.69	85.87	−0.137
BnaA05g28810D	73	8054.75	10.06	28.36	105.34	−0.138
BnaA09g33670D	76	8324.03	10.05	24.56	98.68	−0.150
BnaAnng24190D	76	8144.84	10.06	26.06	102.5	−0.045
BnaC01g39000D	73	8068.82	10.11	21.31	106.71	−0.149
BnaC03g35850D	73	8038.71	10.12	26.88	105.34	−0.116
BnaC04g27370D	743	80,601.76	7.98	36.69	86.42	−0.107
BnaC05g43280D	73	8040.72	10.06	27.2	105.34	−0.138
BnaC06g14570D	76	8144.84	10.06	26.06	102.5	−0.045
BnaCnng30990D	76	8306	10.05	24.56	103.82	−0.125

## Data Availability

The data presented in this study can be obtained from the corresponding author upon reasonable request.

## Supplementary Materials

The following supporting information can be downloaded at https://www.mdpi.com/article/10.3390/plants15121808/s1. Table S1: Sequences of RT-qPCR primers used in this study. Table S2: Full-length coding sequences of the identified *BnaS1fa* family genes in *B. napus*. Table S3: Gene annotation information of the *BnaS1fa* family genes. Table S4: Predicted subcellular localization of the BnaS1fa proteins. Figure S1: multiple sequence alignment.
