# Supplementary figures and images for: Genome-Wide Identification of S1fa Transcription Factors in Brassica napus and Screening of Key Genes BnaS1fa9 and BnaS1fa10 Responsive to Salt, Heat and Cold Stresses
Source: Plants (Basel). 2026 Jun 12;15(12):1808. doi: 10.3390/plants15121808 (PMC13306340; doi:10.3390/plants15121808)

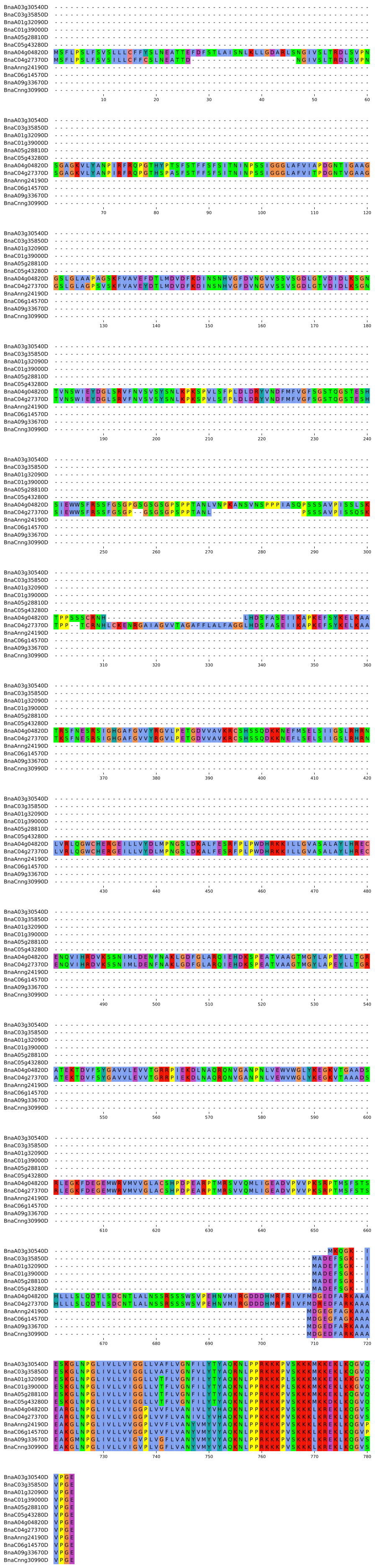

Supplement: Supplementary file 1 [file plants-15-01808-s001.zip › Figure S1.png]
